# Protein Kinases as Mediators for miRNA Modulation of Neuropathic Pain

**DOI:** 10.3390/cells14080577

**Published:** 2025-04-11

**Authors:** Leah Chang, Zala Čok, Lei Yu

**Affiliations:** Department of Genetics, Center of Alcohol & Substance Use Studies, Rutgers University, Piscataway, NJ 08854, USA; lc1100@scarletmail.rutgers.edu (L.C.);

**Keywords:** miRNAs, kinases, neuropathic pain

## Abstract

Neuropathic pain is a chronic condition resulting from injury or dysfunction in the somatosensory nervous system, which leads to persistent pain and a significant impairment of quality of life. Research has highlighted the complex molecular mechanisms that underlie neuropathic pain and has begun to delineate the roles of microRNAs (miRNAs) in modulating pain pathways. miRNAs, which are small non-coding RNAs that regulate gene expression post-transcriptionally, have been shown to influence key cellular processes, including neuroinflammation, neuronal excitability, and synaptic plasticity. These processes contribute to the persistence of neuropathic pain, and miRNAs have emerged as critical regulators of pain behaviors by modulating signaling pathways that control pain sensitivity. miRNAs can influence neuropathic pain by targeting genes that encode protein kinases involved in pain signaling. This review focuses on miRNAs that have been demonstrated to modulate neuropathic pain behavior through their effects on protein kinases or their immediate upstream regulators. The relationship between miRNAs and neuropathic pain behaviors is characterized as either an upregulation or a downregulation of miRNA levels that leads to a reduction in neuropathic pain. In the case of miRNA upregulation resulting in an alleviation of neuropathic pain behaviors, protein kinases exhibit a positive correlation with neuropathic pain, whereas decreased protein kinase levels correlate with diminished neuropathic pain behaviors. The only exception is GRK2, which shows an inverse correlation with neuropathic pain. In the case of miRNA downregulation resulting in a reduction in neuropathic pain behaviors, protein kinases display mixed relationships to neuropathic pain, with some kinases exhibiting positive correlation, while others exhibit negative correlation. By exploring how protein kinases mediate miRNA modulation of neuropathic pain, valuable insight may be gained into the pathophysiology of neuropathic pain, offering potential therapeutic targets for developing more effective strategies for pain management.

## 1. Introduction

Neuropathic pain is a chronic, debilitating condition caused by injury or dysfunction of the somatosensory nervous system [1,2,3,4,5]. Unlike nociceptive pain, which stems from acute tissue damage and typically resolves with healing, neuropathic pain persists beyond the initial injury, often becoming long-term or even lifelong. Affecting an estimated 3% to 17% of the global population [6,7], neuropathic pain significantly diminishes quality of life and remains a major challenge within the field of pain management [8,9,10,11].

Despite decades of research, effective treatment of neuropathic pain remains a significant clinical challenge [11,12,13,14,15]. Current pharmaceutical approaches, including antiepileptic drugs, antidepressants, and opioids, often fall short, with nearly 50% of patients failing to achieve adequate pain relief [12,13,14,16,17]. This underscores the urgent need for novel therapeutic strategies. A major obstacle lies within the complex molecular and cellular mechanisms underlying neuropathic pain, which involve neuroinflammation, altered synaptic plasticity, and dysregulated signaling pathways [4,18,19]. A deeper understanding of these processes at the molecular level is essential for identifying new and more effective therapeutic targets.

MicroRNAs (miRNAs) are a class of short, non-coding RNA molecules that regulate gene expression post-transcriptionally by binding to target messenger RNAs (mRNAs), leading to their degradation or translational repression [20,21,22,23]. These molecules play essential roles in a variety of biological and pathological processes, including neuroinflammation, neuronal excitability, and synaptic plasticity—factors that contribute to the persistence of neuropathic pain [24,25]. Recent evidence suggests that miRNAs are key modulators of neuropathic pain behaviors, either exacerbating or alleviating pain states by targeting specific signaling pathways.

Among these pathways, protein kinases are critical cellular regulators that mediate diverse biological functions through the phosphorylation of proteins [26,27,28]. Many protein kinases have been implicated in neuropathic pain modulation, either promoting or suppressing pain signaling depending on their specific roles in various intracellular cascades [29,30]. Given the emerging evidence linking miRNA activity to modified protein kinase signaling in neuropathic pain models, this review focuses on miRNAs that have been demonstrated to modulate neuropathic pain behavior through direct or indirect effects on the mRNAs encoding protein kinases or their immediate upstream regulators. By elucidating these interactions, we aim to provide insight into the regulatory mechanisms of neuropathic pain and potential therapeutic targets. For a more comprehensive list of miRNAs implicated in neuropathic pain, readers are directed to the following review articles [31,32,33,34,35,36,37].

## 2. miRNA Modulation: Upregulation or Downregulation Can Alleviate Neuropathic Pain

A growing body of literature has shown that miRNAs can causally influence neuropathic pain behaviors. We identified two distinct motifs regarding the relationships that characterize the interconnection between changes in miRNA levels and the consequences on neuropathic pain states.

**Figure 1:**
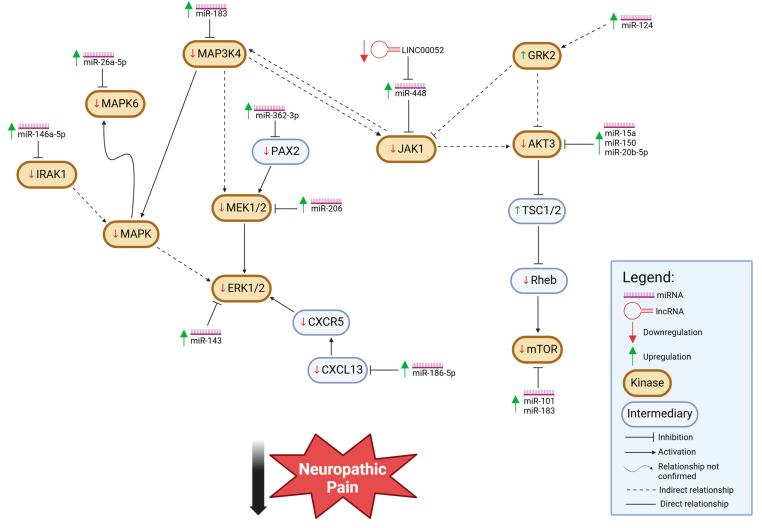
Upregulation of miRNA, via kinase mediation, alleviates neuropathic pain. The upregulation of miRNAs is indicated by an upward green arrow. Reduction in kinase activities or expression levels, which is the case for all kinases directly or indirectly impacted by miRNA, is marked by a downward red arrow, except for GRK2, which shows increased levels with miRNA upregulation with an upward green arrow. Additionally, TSC1/2, an intermediary protein, is also upregulated in this motif. The outcome of all indicated changes in miRNA or other factors’ levels is the alleviation of neuropathic pain.

For the first motif, which is more abundantly reported in the literature, the upregulation of specific miRNAs results in the attenuation of neuropathic pain behaviors; in other words, the increased expression of these miRNAs leads to reduced pain. As summarized in Table 1 and illustrated in Figure 1, the miRNAs belonging to this relationship pattern predominantly target protein kinases that exhibit a positive correlation with neuropathic pain; specifically, in this model, when the levels of these protein kinases decrease, neuropathic pain behaviors also diminish. This suggests that these kinases play a pro-nociceptive role in neuropathic pain at the cellular level, and their suppression, whether by miRNAs or other means, may provide therapeutic benefits. However, there is one exception to this pattern of parallel change between the level of kinase activity and the severity of neuropathic pain, G-protein receptor kinase 2 (GRK2). This kinase displays a negative correlation with neuropathic pain [38], with GRK2 levels increasing with miRNA expression as neuropathic pain subsides.

**Figure 2:**
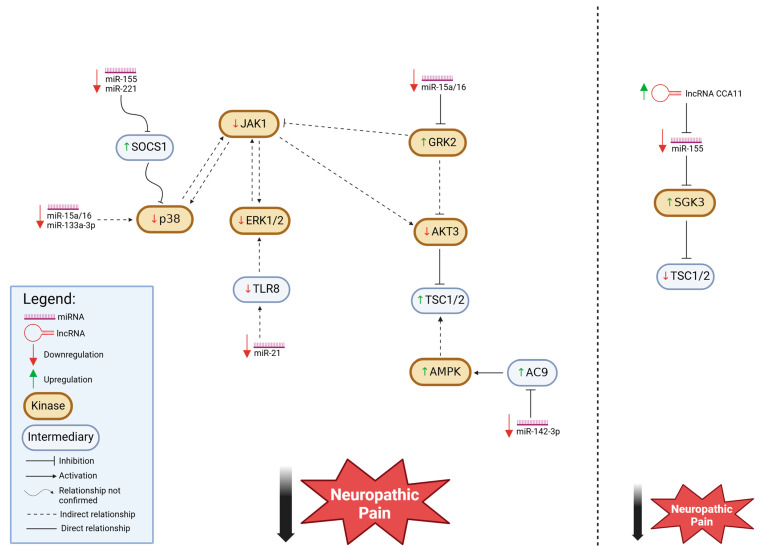
Downregulation of miRNA, via kinase mediation, alleviates neuropathic pain. Downregulation of miRNAs is indicated by a downward red arrow. Changes in the levels of kinases and/or other factors can be either increased, as indicated by an upward green arrow, or decreased, as indicated by a downward red arrow. Note: the SGK3-mediated pathway is distinct from the indicated kinase network due to its opposing effect on TSC1/2 compared to AKT3, which is another upstream regulator of TSC1/2. Depending on the upstream regulator involved, TSC1/2 expression can be either upregulated or downregulated, correlating with pain attenuation. Ultimately, the combined effects of changes in miRNA levels and other regulatory factors contribute to the reduction in neuropathic pain.

For the second motif, the downregulation of certain miRNAs results in a reduction in neuropathic pain behaviors. As summarized in Table 2 and illustrated in Figure 2, the kinases targeted by the miRNAs within this pattern display mixed relationships to neuropathic pain, with some kinases exhibiting a positive correlation in which kinase downregulation relieves pain, and other kinases exhibiting a negative correlation in which kinase downregulation exacerbates pain. This suggests a more complex regulatory landscape in which the miRNA-mediated modulation of protein kinases has varying effects depending on the specific molecular context.

Table 1 and Table 2 list the miRNA–kinase–neuropathic pain cases, indicating the directionality of changes for miRNAs, kinases, and neuropathic pain behaviors. Table 3 lists the kinase names with their abbreviations discussed in this article, as well as alternative abbreviations often encountered in the literature. By categorizing these patterns of miRNA involvement in neuropathic pain, we can begin to discern relevant regulatory mechanisms and potential therapeutic targets. The following sections will explore specific miRNA-protein kinase interactions, highlighting the functional roles of protein kinases in neuropathic pain and implications for future research and clinical applications.

## 3. Protein Kinase Involvement in miRNA Upregulation Leading to Alleviation of Neuropathic Pain

Multiple kinases, through direct or indirect interactions, can form a complex regulatory network (Figure 1). A general pattern within this network reveals that kinase activity is co-regulated with the attenuation of neuropathic pain; specifically, key kinases are downregulated in parallel with reductions in pain behavior. This coordinated downregulation suggests a functional relationship between these kinases and pain modulation. Importantly, miRNAs play a crucial role in this regulatory framework, as their upregulation in these cases contributes to the suppression of specific kinases involved in pain signaling. By targeting and downregulating these kinases, upregulated miRNAs help reinforce the pathway leading to neuropathic pain relief. This intricate interplay between miRNA expression and kinase regulation highlights a potential avenue for therapeutic intervention in neuropathic pain management.

### 3.1. Mitogen-Activated Protein Kinase Kinase Kinase 4 (MAP3K4)

Mitogen-activated protein kinase kinase kinase 4 (MAP3K4) plays a crucial role in cellular signaling, particularly in the mitogen-activated protein kinase (MAPK) cascade [60,61,62]. In this cascade, it is upstream from many kinases such as MKK4/6/7, JNK, and p38 [60,61,62].

MAP3K4 activates the MAPK pathway by phosphorylating and activating MAP2Ks [60,61,62], specifically MKK4 and MKK7 [62], which are responsible for phosphorylating and activating downstream MAPKs such as JNK and p38 [63,64]; therefore, MAP3K4 serves as a key regulator of the MAPK signaling cascade.

MAP3K4 indirectly [60,61,62] influences MEK1/2 through cross-talk between the JNK/p38 MAPK pathways and the ERK pathway [60,65,66,67,68]. Additionally, MAP3K4 is an upstream kinase in the MAPK pathway, triggering the cascade, which eventually leads to MEK1/2 activation [62,69].

Cross-talk between JAK1 and MAP3K4 occurs through the activation of MAPK pathways [60,70]. JAK1-STAT signaling can influence the activation of MAPK cascades indirectly by promoting the expression of genes that regulate proteins involved in MAPK activation [71]. Specifically, MAP3K4 can activate JNK [62,72], which can contribute to the inflammatory response initiated by JAK1 activation [73,74,75]. This cross-talk between cytokine signaling and MAPK pathways defines the complex relationship between the two kinases.

In the CCI model of rats, increased miR-183 levels suppressed MAP3K4 activities, resulting in attenuated neuropathic pain [39].

### 3.2. AKT Serine/Threonine Kinase 3 (AKT3)

AKT serine/threonine kinase 3 (AKT3) is a key regulator in the phosphoinositide 3-kinase (PI3K)-AKT-mTOR pathway, influencing cell growth, survival, and metabolism, particularly in brain development and neuronal protection [76].

In the neuropathic pain model of CCI, increased levels of miR-15a [40], miR-150 [41], and miR-20b-5p [42] suppressed AKT3 kinase activities, leading to attenuated neuropathic pain.

### 3.3. Mechanistic Target of Rapamycin Kinase (mTOR)

The mechanistic target of rapamycin kinase (mTOR) mediates cellular responses to stressors such as DNA damage and nutrient deprivation [77]. mTOR is a component of two distinct complexes: mechanistic target of rapamycin complex 1 (mTORC1), which controls protein synthesis, cell growth, and proliferation [78], and mechanistic target of rapamycin complex 1 (mTORC2), which is a regulator of the actin cytoskeleton and promotes cell survival and cell cycle progression [78].

AKT3 promotes mTOR activation by inhibiting tuberous sclerosis complex subunit 1/2 (TSC1/2) through phosphorylation [79,80,81,82]. This, in turn, allows Ras homolog enriched in brain (Rheb) to remain active and stimulate mTOR signaling [83,84]; thus, the downregulation of AKT3 positively correlates with mTOR downregulation and neuropathic pain attenuation.

In the neuropathic pain model of CCI, increased levels of miR-101 [43] and miR-183 [44] suppressed mTOR expression, attenuating neuropathic pain.

### 3.4. Mitogen-Activated Protein Kinase 1/2 (MEK1/2)

Mitogen-activated protein kinase 1/2 (MEK1/2) is a dual-specificity kinase that functions as a critical component of the MAPK/ERK signaling pathway [85]. They act as intermediaries between MAP3Ks, such as MAP3K4, by phosphorylating and activating ERK1/2 [86], which in turn regulates cell proliferation, differentiation, and survival [87].

MAP3K4 is upstream of MEK1/2 in certain signaling cascades [88], and activates MEK1/2 through intermediates such as MAP2Ks [86], contributing to ERK1/2 activation.

MEK1/2 are the direct activators of ERK1/2 [85]. Upon activation by upstream kinases, MEK1/2 phosphorylates ERK1/2 on specific threonine and tyrosine residues, leading to ERK1/2 activation and subsequent cellular responses [89].

Paired-box gene 2 (PAX2) is a transcription factor that promotes the expression of upstream activators such as receptor tyrosine kinases (RTKs) [90,91], leading to the activation of Ras and then Raf, ultimately leading to the phosphorylation and activation of MEK1/2 [92].

In the SCI rat model, increased levels of miR-362-3p suppress PAX2, which in turn leads to the suppression of MEK1/2 and, therefore, reduced neuropathic pain levels [45]; similarly, in the model of CCI of DRG in rats, increased miR-206 suppresses MEK and neuropathic pain [46], though it is not indicated that PAX2 is involved in this mechanism, nor is the specific isoform of MEK specified.

### 3.5. Extracellular Signal-Regulated Kinase 1/2 (ERK1/2)

Extracellular signal-regulated kinase (ERK1/2) is located downstream from MEK in the MAPK cascade and is a key enzyme in the ERK signaling pathway [88]. MEK1/2 phosphorylates ERK1/2 on threonine (T) and tyrosine (Y) residues within the threonine-glutamic acid-tyrosine (TEY) motif [93], activating ERK1/2.

Upon activation, toll-like receptor 8 (TLR8) recruits myeloid differentiation primary response 88 (MyD88) [94] which facilitates the activation of another protein kinase, IRAK1 [94,95,96], associates with the tumor necrosis factor (TNF) receptor-associated factor 6 (TRAF6) [97], aiding in the activation of MAPK signaling. This, in turn, leads to the activation of MEK1/2, which then phosphorylates and activates ERK1/2.

C-X-C motif chemokine ligand 13 (CXCL13) is a chemokine that binds to the C-X-C motif chemokine receptor 5 (CXCR5) [98], which is a G-protein-coupled receptor (GPCR). Upon CXCL13 activation, CXCR5 activates G-protein signaling, leading to MEK1/2 activation [99], which leads to ERK1/2 phosphorylation and activation [95].

In the neuropathic pain model of SNL-induced DRG of rats, increased levels of miR-143 [48] suppressed ERK1/2 activity, leading to attenuated neuropathic pain; similarly, in the neuropathic pain model of SNL in mice, increased miR-186-5p [47] suppressed CXCL13 and subsequently suppressed CXCR5 and ERK, leading to attenuated neuropathic pain. In this specific model, though, it is not specified which isoform of ERK is suppressed, but rather, the general type of kinase, ERK, is identified.

### 3.6. Mitogen-Activated Protein Kinase 6 (MAPK6)

Mitogen-activated protein kinase 6 (MAPK6) is a non-canonical MAPK, meaning it has unique activation mechanisms and does not follow the traditional rapidly accelerated fibrosarcoma (RAF)-MEK-ERK cascade [100,101,102]. Unlike ERK1/2′s activation through the TEY motif, MAPK6′s corresponding motif is SEG [100,103,104,105]. Not much is known about the role of MAPK6, but it does engage in regulatory relationships within the broad MAPK signaling network [106,107]. MAPK6 is activated through protein phosphorylation cascades and acts as an integration point for multiple biochemical signals [104,107].

In the neuropathic pain model of CCI, the upregulation of miR-26a-5p suppressed MAPK6 expression, resulting in attenuated neuropathic pain [49].

### 3.7. Interleukin 1 Receptor-Associated Kinase (IRAK1)

Interleukin 1 receptor-associated kinase (IRAK1) plays an important role in the regulation of the expression of inflammatory genes. Upon activation by their respective ligands, toll-like receptors (TLRs) and interleukin-1 (IL-1) recruit MyD88 [97], leading to the phosphorylation and activation of IRAK1 [96,108]. Once activated, IRAK1 phosphorylates and activates TRAF6, which then activates several kinases in the MAPK family, including ERK1/2, p38, and JNK [94].

In the neuropathic pain model of CCI, increased expression of miR-146a-5p suppressed IRAK1 signaling, leading to attenuated neuropathic pain [50].

### 3.8. Janus Kinase 1 (JAK1)

Janus kinase 1 (JAK1) is a protein that helps transmit signals for cytokines and growth factors [109], making it a key part of immune function. JAK1 is activated by cytokines, stimulating phosphoinositide 3-kinase (PI3K), which in turn activates AKT3 [109].

In the neuropathic pain model of SNL in rats, decreased levels of the lncRNA (long non-coding RNA) LINC00052 (long intergenic non-coding RNA 00052) have been found to increase levels of miR-448 [51], leading to decreased JAK1 levels and attenuated neuropathic pain.

### 3.9. G Protein-Coupled Receptor Kinase 2 (GRK2)

G protein-coupled receptor kinase 2 (GRK2) plays a key role in GPCR signaling [110,111], indirectly inhibiting JAK1 by phosphorylating upstream GPCRs [112,113,114], which disrupts the JAK signaling pathway and thereby reducing the downstream signaling cascade associated with JAK1 activity [112,115].

GRK2 negatively regulates AKT3 signaling through interactions with phosphatases such as protein phosphatase 2A (PP2A), leading to AKT3 dephosphorylation and, therefore, inactivation [110,111].

In the neuropathic pain model of de novo GRK2 knockout mice, increased levels of miR-124 [38] increased GRK2 expression, leading to attenuated neuropathic pain.

## 4. Protein Kinase Involvement in miRNA Downregulation Leading to Alleviation of Neuropathic Pain

Figure 2 illustrates the protein kinases involved in the second motif, along with their direct and indirect interaction partners. This kinase network presents a more intricate regulatory landscape in the context of neuropathic pain behaviors. Unlike the uniform co-regulation observed in other networks, kinases in these cases show a more nuanced interplay, where some kinases exhibit a positive correlation with neuropathic pain, meaning their upregulation is associated with increased pain behaviors, while others display a negative correlation in which their downregulation aligns with pain attenuation. These opposing regulatory pathways suggest that different subsets of kinases may contribute to distinct mechanisms underlying pain modulation, potentially reflecting the balance between pro-inflammatory and anti-inflammatory signaling pathways. Understanding these complex interactions may provide deeper insight into the molecular underpinnings of neuropathic pain and inform the development of more targeted therapeutic strategies.

### 4.1. Mitogen-Activated Protein Kinase (MAPK): p38

Mitogen-activated protein kinase (MAPK) is a protein kinase family that controls how cells respond to stimuli [104]. p38 is part of the MAPK family and regulates many important cellular processes [116] such as inflammation, cell growth, apoptosis, and tissue homeostasis [117]. Four p38 isoforms have been identified (p38α, p38β, p38γ, and p38δ) [117], though it is not indicated which isoform specifically is involved in the neuropathic pain network.

JAK1 and MAPK pathways interact in a complex but coordinated manner. JAK1, through cytokine receptor activation [118,119], can indirectly activate MAPK signaling [120,121]; additionally, MAPK signaling can modulate JAK1 activity [122,123], creating feedback loops that fine-tune cellular functions.

Suppressor of cytokine signaling 1 (SOCS1) inhibits MAPK signaling by indirectly blocking the activation of the upstream kinases, primarily by targeting and inhibiting the JAK family of kinases, which are crucial for the phosphorylation cascade leading to MAPK activation [124].

In the CCI model of rats, decreased levels of both miR-155 [52] and miR-221 [53] led to increased levels of SOCS1. This decreased p38 expression levels, attenuating neuropathic pain.

In the diabetes mellitus (DM) sciatic nerve of model rats, decreased levels of miR-133-3p [54] suppressed p38, attenuating neuropathic pain; additionally, in the CCI model of mice, decreased levels of miR-15a/16 [55] also suppressed p38, attenuating neuropathic pain.

### 4.2. Extracellular Signal-Regulated Kinase (ERK)

Extracellular signal-regulated kinase (ERK) is located downstream from MEK in the MAPK cascade and is a key enzyme within the ERK signaling pathway [88].

In the DRG Tlr8 knockout mouse model, decreased expression of miR-21 [56] downregulated Tlr8 and, consequently, led to decreased levels of ERK as well as attenuated neuropathic pain.

### 4.3. Adenosine Monophosphate-Activated Protein Kinase (AMPK)

Adenosine monophosphate-activated protein kinase (AMPK) is a protein kinase that plays a crucial role in regulating energy metabolism [125]. AMPK and AKT3 both work on TSC1/2 with opposing effects. AMPK activates TSC1/2, which leads to mTOR inhibition [126,127], while AKT3 inhibits TSC1/2, which leads to mTOR activation [128,129]. This balance between AMPK and AKT3 through TSC1/2 ensures regulated cellular responses.

In the CCI SNI model of rats, decreased levels of miR-142-3p led to an increase in AC9 levels, which in turn led to a decrease in cyclic AMP (cAMP) [57]. This decrease in cAMP increased AMPK expression, attenuating neuropathic pain.

### 4.4. Serum/Glucocorticoid Regulated Kinase Family Member 3 (SGK3)

Serum/glucocorticoid regulated kinase family member 3 (SGK3) phosphorylates several target proteins and has a role in neutral amino acid transport and activation of potassium and chloride channels [130,131]. SGK3 phosphorylates TSC, inhibiting TSC1/2 [132,133]. This inhibition leads to the activation of Rheb [134,135], which stimulates mTOR [133,134].

In the neuropathic pain models of bilateral CCI, upregulation of lncRNA CCA11 [58] suppressed miR-155 [59], upregulating SGK3 and attenuating neuropathic pain.

### 4.5. G Protein-Coupled Receptor Kinase 2 (GRK2)

G protein-coupled receptor kinase 2 (GRK2) plays a key role in GPCR signaling [110,111]. GRK2 negatively regulates AKT3 signaling through interactions with phosphatases such as protein phosphatase 2A (PP2A) [136,137,138], leading to AKT3 dephosphorylation and inactivation.

In the CCI model of mice, decreased levels of miR-15a/16 [55] led to increased GRK2 expression, attenuating neuropathic pain.

## 5. Concluding Remarks

In this review, we summarize key findings from the literature demonstrating causal relationships between miRNA regulation and neuropathic pain behaviors, with a specific focus on protein kinases as mediators of these effects. Two relationship patterns characterize miRNA modulation of neuropathic pain: (1) upregulation of miRNA attenuates neuropathic pain, largely through the suppression of pro-nociceptive protein kinases, and (2) downregulation of miRNA leads to pain attenuation, either by relieving the receptive regulation of anti-neuropathic signaling pathways, or by directly promoting their activation. These relationship patterns highlight the complex regulatory networks underlying the mechanisms for neuropathic pain, where protein kinases serve as critical molecular mediators in the cellular signaling cascades.

Given the high prevalence of neuropathic pain and a lack of effective therapies [6,7,9,10,11,12,13,14,15,16,17], there is an urgent need to develop novel treatment strategies that target the underlying molecular mechanisms of pain pathophysiology. Our discussion underscores the potential of miRNA-based approaches in this context, as miRNAs serve as upstream regulators and are capable of modulating multiple pain-related pathways simultaneously. The role of protein kinases as mediators of miRNA also points to the potential of targeting intracellular signaling molecules as a pain management strategy, offering the opportunity to identify kinase-specific interventions that may work independently or synergistically with miRNA-targeted therapies.

The therapeutic potential of miRNA modulation is increasingly being recognized [139,140,141]; specifically, both miRNA mimics and miRNA inhibitors can be used to manipulate miRNA levels, thus achieving the in vivo effect of either miRNA upregulation or miRNA downregulation [142,143,144]. In the context of neuropathic pain, the evidence summarized in this review suggests that both miRNA activation and inhibition could have therapeutic value, depending on the specific miRNA and its downstream targets. For miRNAs whose upregulation can lead to alleviation of neuropathic pain (Table 1, Figure 1), desirable therapeutic outcomes may be achieved with miRNA mimics, i.e., synthetic double-stranded RNA molecules that mimic the function of these endogenous miRNAs; similarly, for miRNAs whose downregulation can attenuate neuropathic pain (Table 2, Figure 2), favorable clinical results may be attained with miRNA inhibitors, i.e., single-stranded RNAs that are complementary to endogenous miRNAs, thus achieving the effect of gene silencing by specifically inhibiting these endogenous miRNAs. The ability to selectively regulate miRNA activity using miRNA modulators may open exciting avenues for the development of precision medicine approaches to neuropathic pain management.

## Figures and Tables

**Table 1:** Upregulation of miRNA, via kinase mediation, alleviates neuropathic pain.

↑ miRNA ⇒ Kinase ⇒ ↓ Neuropathic Pain						
lncRNA	miRNA	Intermediary	Protein Kinase	Neuropathic Pain	Model	Reference
-	miR-183 ↑	-	MAP3K4 ↓	down ↓	Rat, CCI	[39]
-	miR-15a ↑	-	AKT3 ↓	down ↓	Rat, CCI	[40]
-	miR-150 ↑	-	AKT3 ↓	down ↓	Rat, CCI	[41]
-	miR-20b-5p ↑	-	AKT3 ↓	down ↓	Rat, CCI	[42]
-	miR-101 ↑	-	mTOR ↓	down ↓	Rat, CCI	[43]
-	miR-183 ↑	-	mTOR ↓	down ↓	Rat, CCI	[44]
-	miR-362-3p ↑	PAX2 ↓	MEK1/2 ↓	down ↓	Rat, SCI	[45]
-	miR-206 ↑	-	MEK ↓	down ↓	Rat, CCI DRG	[46]
-	miR-186-5p ↑	CXCL13 ↓ CXCR5 ↓	ERK ↓	down ↓	Mouse, SNL	[47]
-	miR-143 ↑	-	ERK1/2 ↓	down ↓	Rat, SNL-induced DRG	[48]
-	miR-26a-5p ↑	-	MAPK6 ↓	down ↓	Rat, CCI	[49]
-	miR-146a-5p ↑	-	IRAK1 ↓	down ↓	Rat, CCI	[50]
LINC00052 ↓	miR-448 ↑	-	JAK1 ↓	down ↓	Rat, SNL	[51]
-	miR-124 ↑	-	GRK2 ↑	down ↓	Mouse, de novo GRK2 knockout	[38]

Abbreviations of nerve injury models: CCI, chronic constriction injury; DRG, dorsal root ganglia; SCI, spinal cord injury; SNL, spinal nerve ligation. Upward arrows (↑) indicate upregulation; downward arrows (↓) indicate downregulation.

**Table 2:** Downregulation of miRNA, via kinase mediation, alleviates neuropathic pain.

↓ miRNA ⇒ Kinase ⇒ ↓ Neuropathic Pain						
lncRNA	miRNA	Intermediary	Protein Kinase	Neuropathic Pain	Model	Reference
-	miR-155 ↓	SOCS1 ↑	p38 ↓	down ↓	Rat, CCI	[52]
-	miR-221 ↓	SOCS1 ↑	p38 ↓	down ↓	Rat, CCI	[53]
-	miR-133a-3p ↓	-	p38 ↓	down ↓	Rat (diabetic), sciatic nerve	[54]
-	miR-15a/16 ↓	-	p38 ↓	down ↓	Mouse, CCI	[55]
-	miR-21 ↓	TLR8 ↓	ERK ↓	down ↓	Mouse, DRG, Tlr8 knockout	[56]
-	miR-142-3p ↓	AC9 ↑	AMPK ↑	down ↓	Rat, CCI, SNI	[57]
lncRNA CCA11 ↑	miR-155 ↓	-	SGK3 ↑	down ↓	Rat, bilateral CCI	[58]
-	miR-155 ↓	-	SGK3 ↑	down ↓	SD rat, bilateral CCI	[59]
-	miR-15a/16 ↓	-	GRK2 ↑	down ↓	Mouse, CCI	[55]

Abbreviations of nerve injury models: CCI, chronic constriction injury; DRG, dorsal root ganglia; SD rat, Sprague Dawley rat; SNI, spared nerve injury. Upward arrows (↑) indicate upregulation; downward arrows (↓) indicate downregulation.

**Table 3:** Protein kinase names and abbreviations.

Protein Kinase Abbreviation	Alternative Abbreviations	Full Name
MAP3K4	MTK1; MEKK4; MAPKKK4; PRO0412; MKKK4	Mitogen-activated protein kinase kinase kinase 4
JNK	MAPK8; JNK1; PRKM8; SAPK1; JNK-46; JNK1A2; SAPK1c; JNK21B1/2	Jun N-terminal kinase
MKK4	MAP2K4; JNKK; MEK4; SEK1; SKK1; JNKK1; SERK1; MAPKK4; PRKMK4; SAPKK1	Mitogen-activated protein kinase kinase 4
MEK1/2	MAP2K1/2	Mitogen-activated protein kinase 1/2
ERK1/2	MAPK1/2	Extracellular signal-regulated kinase 1/2
JAK1	JTK3; AIIDE; JAK1A; JAK1B	Janus kinase 1
MAP2K	MKK	Mitogen-activated protein kinase kinase
AKT3	MPPH; PKBG; MPPH2; PRKBG; STK-2; PKB-GAMMA; RAC-gamma; RAC-PK-gamma	AKT Serine/Threonine kinase
mTOR	SKS; FRAP; FRAP1; FRAP2; RAFT1; RAPT1	Mechanistic target of rapamycin kinase
IRAK1	IRAK; pelle	Interleukin 1 receptor-associated kinase
MAPK6	ERK3; PRKM6; p97MAPK; HsT17250	Mitogen-activated protein kinase 6
GRK2	BARK1; ADRBK1; BETA-ARK1	G protein-coupled receptor kinase 2
AMPK	PRKAA1; AMPKa1; AMPK alpha 1	Adenosine monophosphate-activated protein kinase
SGK3	CISK; SGK2; SGKL	Serum/glucocorticoid-regulated kinase family member 3

## Data Availability

No new data were created or analyzed in this study.

## References

[1] Colloca L., Ludman T., Bouhassira D., Baron R., Dickenson A.H., Yarnitsky D., Freeman R., Truini A., Attal N., Finnerup N.B. (2017). Neuropathic pain. Nat. Rev. Dis. Primers.

[2] Scholz J., Finnerup N.B., Attal N., Aziz Q., Baron R., Bennett M.I., Benoliel R., Cohen M., Cruccu G., Davis K.D. (2019). The IASP classification of chronic pain for ICD-11: Chronic neuropathic pain. Pain.

[3] Leone C.M., Truini A. (2024). Understanding neuropathic pain: The role of neurophysiological tests in unveiling underlying mechanisms. J. Anesth. Analg. Crit. Care.

[4] Ma Y.C., Kang Z.B., Shi Y.Q., Ji W.Y., Zhou W.M., Nan W. (2024). The Complexity of Neuropathic Pain and Central Sensitization: Exploring Mechanisms and Therapeutic Prospects. J. Integr. Neurosci..

[5] Lima Pessôa B., Hauwanga W.N., Thomas A., Valentim G., McBenedict B. (2024). A Comprehensive Narrative Review of Neuropathic Pain: From Pathophysiology to Surgical Treatment. Cureus.

[6] van Hecke O., Austin S.K., Khan R.A., Smith B.H., Torrance N. (2014). Neuropathic pain in the general population: A systematic review of epidemiological studies. Pain.

[7] Cavalli E., Mammana S., Nicoletti F., Bramanti P., Mazzon E. (2019). The neuropathic pain: An overview of the current treatment and future therapeutic approaches. Int. J. Immunopathol. Pharmacol..

[8] Bouhassira D., Lanteri-Minet M., Attal N., Laurent B., Touboul C. (2008). Prevalence of chronic pain with neuropathic characteristics in the general population. Pain.

[9] Baron R., Binder A., Wasner G. (2010). Neuropathic pain: Diagnosis, pathophysiological mechanisms, and treatment. Lancet Neurol..

[10] Attal N., Bouhassira D., Baron R. (2018). Diagnosis and assessment of neuropathic pain through questionnaires. Lancet Neurol..

[11] Feldman A., Weaver J. (2025). Pharmacologic and Nonpharmacologic Management of Neuropathic Pain. Semin. Neurol..

[12] Dworkin R.H., O’Connor A.B., Backonja M., Farrar J.T., Finnerup N.B., Jensen T.S., Kalso E.A., Loeser J.D., Miaskowski C., Nurmikko T.J. (2007). Pharmacologic management of neuropathic pain: Evidence-based recommendations. Pain.

[13] O’Connor A.B., Dworkin R.H. (2009). Treatment of neuropathic pain: An overview of recent guidelines. Am. J. Med..

[14] Dworkin R.H., O’Connor A.B., Kent J., Mackey S.C., Raja S.N., Stacey B.R., Levy R.M., Backonja M., Baron R., Harke H. (2013). Interventional management of neuropathic pain: NeuPSIG recommendations. Pain.

[15] Andrejic N., Božovic I., Moradi H., Tataei R., Knezevic N.N. (2025). Neuropathic pain management: A focused review of current treatments and novel data from main ongoing clinical trials. Expert. Opin. Investig. Drugs.

[16] Sadegh A.A., Gehr N.L., Finnerup N.B. (2024). A systematic review and meta-analysis of randomized controlled head-to-head trials of recommended drugs for neuropathic pain. Pain. Rep..

[17] Moisset X. (2024). Neuropathic pain: Evidence based recommendations. Presse Med..

[18] Pacifico P., Menichella D.M. (2024). Molecular mechanisms of neuropathic pain. Int. Rev. Neurobiol..

[19] Rugnath R., Orzechowicz C., Newell C., Carullo V., Rugnath A. (2024). A Literature Review: The Mechanisms and Treatment of Neuropathic Pain-A Brief Discussion. Biomedicines.

[20] Bartel D.P. (2004). MicroRNAs: Genomics, biogenesis, mechanism, and function. Cell.

[21] Filipowicz W., Bhattacharyya S.N., Sonenberg N. (2008). Mechanisms of post-transcriptional regulation by microRNAs: Are the answers in sight?. Nat. Rev. Genet..

[22] Guo H., Ingolia N.T., Weissman J.S., Bartel D.P. (2010). Mammalian microRNAs predominantly act to decrease target mRNA levels. Nature.

[23] Suzuki H.I. (2023). Roles of MicroRNAs in Disease Biology. JMA J..

[24] Bredy T.W., Lin Q., Wei W., Baker-Andresen D., Mattick J.S. (2011). MicroRNA regulation of neural plasticity and memory. Neurobiol. Learn. Mem..

[25] Kalpachidou T., Kummer K.K., Kress M. (2020). Non-coding RNAs in neuropathic pain. Neuronal Signal.

[26] Cohen P. (2001). The role of protein phosphorylation in human health and disease. The Sir Hans Krebs Medal Lecture. Eur. J. Biochem..

[27] Ardito F., Giuliani M., Perrone D., Troiano G., Lo Muzio L. (2017). The crucial role of protein phosphorylation in cell signaling and its use as targeted therapy (Review). Int. J. Mol. Med..

[28] Houles T., Yoon S.O., Roux P.P. (2024). The expanding landscape of canonical and non-canonical protein phosphorylation. Trends Biochem. Sci..

[29] Ji R.R., Gereau R.W.t., Malcangio M., Strichartz G.R. (2009). MAP kinase and pain. Brain Res. Rev..

[30] Mai L., Zhu X., Huang F., He H., Fan W. (2020). p38 mitogen-activated protein kinase and pain. Life Sci..

[31] Song G., Yang Z., Guo J., Zheng Y., Su X., Wang X. (2020). Interactions Among lncRNAs/circRNAs, miRNAs, and mRNAs in Neuropathic Pain. Neurotherapeutics.

[32] Gada Y., Pandey A., Jadhav N., Ajgaonkar S., Mehta D., Nair S. (2021). New Vistas in microRNA Regulatory Interactome in Neuropathic Pain. Front. Pharmacol..

[33] Jiang M., Wang Y., Wang J., Feng S., Wang X. (2022). The etiological roles of miRNAs, lncRNAs, and circRNAs in neuropathic pain: A narrative review. J. Clin. Lab. Anal..

[34] Morchio M., Sher E., Collier D.A., Lambert D.W., Boissonade F.M. (2023). The Role of miRNAs in Neuropathic Pain. Biomedicines.

[35] Zhao Y.Y., Wu Z.J., Zhu L.J., Niu T.X., Liu B., Li J. (2023). Emerging roles of miRNAs in neuropathic pain: From new findings to novel mechanisms. Front. Mol. Neurosci..

[36] Sampath K.K., Belcher S., Hales J., Thomson O.P., Farrell G., Gisselman A.S., Katare R., Tumilty S. (2023). The role of micro-RNAs in neuropathic pain-a scoping review. Pain. Rep..

[37] Golmakani H., Azimian A., Golmakani E. (2024). Newly discovered functions of miRNAs in neuropathic pain: Transitioning from recent discoveries to innovative underlying mechanisms. Mol. Pain..

[38] Willemen H.L., Huo X.J., Mao-Ying Q.L., Zijlstra J., Heijnen C.J., Kavelaars A. (2012). MicroRNA-124 as a novel treatment for persistent hyperalgesia. J. Neuroinflammation.

[39] Huang L., Wang L. (2020). Upregulation of miR-183 represses neuropathic pain through inhibiton of MAP3K4 in CCI rat models. J. Cell Physiol..

[40] Cai L., Liu X., Guo Q., Huang Q., Zhang Q., Cao Z. (2020). MiR-15a attenuates peripheral nerve injury-induced neuropathic pain by targeting AKT3 to regulate autophagy. Genes. Genomics.

[41] Cai W., Zhang Y., Liu Y., Liu H., Zhang Z., Su Z. (2019). Effects of miR-150 on neuropathic pain process via targeting AKT3. Biochem. Biophys. Res. Commun..

[42] You H., Zhang L., Chen Z., Liu W., Wang H., He H. (2019). MiR-20b-5p relieves neuropathic pain by targeting Akt3 in a chronic constriction injury rat model. Synapse.

[43] Xie T., Zhang J., Kang Z., Liu F., Lin Z. (2020). miR-101 down-regulates mTOR expression and attenuates neuropathic pain in chronic constriction injury rat models. Neurosci. Res..

[44] Xie X., Ma L., Xi K., Zhang W., Fan D. (2017). MicroRNA-183 Suppresses Neuropathic Pain and Expression of AMPA Receptors by Targeting mTOR/VEGF Signaling Pathway. Cell Physiol. Biochem..

[45] Hu Y., Liu Q., Zhang M., Yan Y., Yu H., Ge L. (2019). MicroRNA-362-3p attenuates motor deficit following spinal cord injury via targeting paired box gene 2. J. Integr. Neurosci..

[46] Sun W., Zhang L., Li R. (2017). Overexpression of miR-206 ameliorates chronic constriction injury-induced neuropathic pain in rats via the MEK/ERK pathway by targeting brain-derived neurotrophic factor. Neurosci. Lett..

[47] Jiang B.C., Cao D.L., Zhang X., Zhang Z.J., He L.N., Li C.H., Zhang W.W., Wu X.B., Berta T., Ji R.R. (2016). CXCL13 drives spinal astrocyte activation and neuropathic pain via CXCR5. J. Clin. Investig..

[48] Xu B., Cao J., Zhang J., Jia S., Wu S., Mo K., Wei G., Liang L., Miao X., Bekker A. (2017). Role of MicroRNA-143 in Nerve Injury-Induced Upregulation of Dnmt3a Expression in Primary Sensory Neurons. Front. Mol. Neurosci..

[49] Zhang Y., Su Z., Liu H.L., Li L., Wei M., Ge D.J., Zhang Z.J. (2018). Effects of miR-26a-5p on neuropathic pain development by targeting MAPK6 in in CCI rat models. Biomed. Pharmacother..

[50] Wang Z., Liu F., Wei M., Qiu Y., Ma C., Shen L., Huang Y. (2018). Chronic constriction injury-induced microRNA-146a-5p alleviates neuropathic pain through suppression of IRAK1/TRAF6 signaling pathway. J. Neuroinflammation.

[51] Wang L., Zhu K., Yang B., Cai Y. (2020). Knockdown of Linc00052 alleviated spinal nerve ligation-triggered neuropathic pain through regulating miR-448 and JAK1. J. Cell Physiol..

[52] Tan Y., Yang J., Xiang K., Tan Q., Guo Q. (2015). Suppression of microRNA-155 attenuates neuropathic pain by regulating SOCS1 signalling pathway. Neurochem. Res..

[53] Xia L., Zhang Y., Dong T. (2016). Inhibition of MicroRNA-221 Alleviates Neuropathic Pain Through Targeting Suppressor of Cytokine Signaling 1. J. Mol. Neurosci..

[54] Chang L.L., Wang H.C., Tseng K.Y., Su M.P., Wang J.Y., Chuang Y.T., Wang Y.H., Cheng K.I. (2020). Upregulation of miR-133a-3p in the Sciatic Nerve Contributes to Neuropathic Pain Development. Mol. Neurobiol..

[55] Li T., Wan Y., Sun L., Tao S., Chen P., Liu C., Wang K., Zhou C., Zhao G. (2019). Inhibition of MicroRNA-15a/16 Expression Alleviates Neuropathic Pain Development through Upregulation of G Protein-Coupled Receptor Kinase 2. Biomol. Ther..

[56] Zhang Z.J., Guo J.S., Li S.S., Wu X.B., Cao D.L., Jiang B.C., Jing P.B., Bai X.Q., Li C.H., Wu Z.H. (2018). TLR8 and its endogenous ligand miR-21 contribute to neuropathic pain in murine DRG. J. Exp. Med..

[57] Li X., Wang S., Yang X., Chu H. (2021). miR-142-3p targets AC9 to regulate sciatic nerve injury-induced neuropathic pain by regulating the cAMP/AMPK signalling pathway. Int. J. Mol. Med..

[58] Dou L., Lin H., Wang K., Zhu G., Zou X., Chang E., Zhu Y. (2017). Long non-coding RNA CCAT1 modulates neuropathic pain progression through sponging miR-155. Oncotarget.

[59] Liu S., Zhu B., Sun Y., Xie X. (2015). MiR-155 modulates the progression of neuropathic pain through targeting SGK3. Int. J. Clin. Exp. Pathol..

[60] Takekawa M., Posas F., Saito H. (1997). A human homolog of the yeast Ssk2/Ssk22 MAP kinase kinase kinases, MTK1, mediates stress-induced activation of the p38 and JNK pathways. EMBO J..

[61] Nguyen K., Tran M.N., Rivera A., Cheng T., Windsor G.O., Chabot A.B., Cavanaugh J.E., Collins-Burow B.M., Lee S.B., Drewry D.H. (2022). MAP3K Family Review and Correlations with Patient Survival Outcomes in Various Cancer Types. Front. Biosci..

[62] Huang Y., Wang G., Zhang N., Zeng X. (2024). MAP3K4 kinase action and dual role in cancer. Discov. Oncol..

[63] Tournier C., Dong C., Turner T.K., Jones S.N., Flavell R.A., Davis R.J. (2001). MKK7 is an essential component of the JNK signal transduction pathway activated by proinflammatory cytokines. Genes. Dev..

[64] Ho D.T., Bardwell A.J., Abdollahi M., Bardwell L. (2003). A docking site in MKK4 mediates high affinity binding to JNK MAPKs and competes with similar docking sites in JNK substrates. J. Biol. Chem..

[65] Zheng C.F., Guan K.L. (1993). Cloning and characterization of two distinct human extracellular signal-regulated kinase activator kinases, MEK1 and MEK2. J. Biol. Chem..

[66] Blank J.L., Gerwins P., Elliott E.M., Sather S., Johnson G.L. (1996). Molecular cloning of mitogen-activated protein/ERK kinase kinases (MEKK) 2 and 3. Regulation of sequential phosphorylation pathways involving mitogen-activated protein kinase and c-Jun kinase. J. Biol. Chem..

[67] Gerwins P., Blank J.L., Johnson G.L. (1997). Cloning of a novel mitogen-activated protein kinase kinase kinase, MEKK4, that selectively regulates the c-Jun amino terminal kinase pathway. J. Biol. Chem..

[68] Xu B., Wilsbacher J.L., Collisson T., Cobb M.H. (1999). The N-terminal ERK-binding site of MEK1 is required for efficient feedback phosphorylation by ERK2 in vitro and ERK activation in vivo. J. Biol. Chem..

[69] Shaul Y.D., Seger R. (2007). The MEK/ERK cascade: From signaling specificity to diverse functions. Biochim. Biophys. Acta.

[70] Zhang W., Liu H.T. (2002). MAPK signal pathways in the regulation of cell proliferation in mammalian cells. Cell Res..

[71] Hu Q., Bian Q., Rong D., Wang L., Song J., Huang H.S., Zeng J., Mei J., Wang P.Y. (2023). JAK/STAT pathway: Extracellular signals, diseases, immunity, and therapeutic regimens. Front. Bioeng. Biotechnol..

[72] Chen W., White M.A., Cobb M.H. (2002). Stimulus-specific requirements for MAP3 kinases in activating the JNK pathway. J. Biol. Chem..

[73] Guma M., Firestein G.S. (2012). c-Jun N-Terminal Kinase in Inflammation and Rheumatic Diseases. Open Rheumatol. J..

[74] Jenkins B.J. (2014). Transcriptional regulation of pattern recognition receptors by Jak/STAT signaling, and the implications for disease pathogenesis. J. Interferon Cytokine Res..

[75] Thompson H.J., Lutsiv T. (2023). Natural Products in Precision Oncology: Plant-Based Small Molecule Inhibitors of Protein Kinases for Cancer Chemoprevention. Nutrients.

[76] Guo N., Wang X., Xu M., Bai J., Yu H., Le Z. (2024). PI3K/AKT signaling pathway: Molecular mechanisms and therapeutic potential in depression. Pharmacol. Res..

[77] Soliman G.A. (2013). The role of mechanistic target of rapamycin (mTOR) complexes signaling in the immune responses. Nutrients.

[78] Simcox J., Lamming D.W. (2022). The central moTOR of metabolism. Dev. Cell.

[79] Dan H.C., Ebbs A., Pasparakis M., Van Dyke T., Basseres D.S., Baldwin A.S. (2014). Akt-dependent activation of mTORC1 complex involves phosphorylation of mTOR (mammalian target of rapamycin) by IkappaB kinase alpha (IKKalpha). J. Biol. Chem..

[80] Tee A.R., Sampson J.R., Pal D.K., Bateman J.M. (2016). The role of mTOR signalling in neurogenesis, insights from tuberous sclerosis complex. Semin. Cell Dev. Biol..

[81] Dunkerly-Eyring B.L., Pan S., Pinilla-Vera M., McKoy D., Mishra S., Grajeda Martinez M.I., Oeing C.U., Ranek M.J., Kass D.A. (2022). Single serine on TSC2 exerts biased control over mTORC1 activation mediated by ERK1/2 but not Akt. Life Sci. Alliance.

[82] Cormerais Y., Lapp S.C., Kalafut K.C., Cisse M.Y., Shin J., Stefadu B., Personnaz J., Schrotter S., D’Amore A., Martin E.R. (2024). AKT-mediated phosphorylation of TSC2 controls stimulus- and tissue-specific mTORC1 signaling and organ growth. bioRxiv.

[83] Polchi A., Magini A., Meo D.D., Tancini B., Emiliani C. (2018). mTOR Signaling and Neural Stem Cells: The Tuberous Sclerosis Complex Model. Int. J. Mol. Sci..

[84] Liao W.T., Chiang Y.J., Yang-Yen H.F., Hsu L.C., Chang Z.F., Yen J.J.Y. (2023). CBAP regulates the function of Akt-associated TSC protein complexes to modulate mTORC1 signaling. J. Biol. Chem..

[85] Wang H., Chi L., Yu F., Dai H., Si X., Gao C., Wang Z., Liu L., Zheng J., Ke Y. (2022). The overview of Mitogen-activated extracellular signal-regulated kinase (MEK)-based dual inhibitor in the treatment of cancers. Bioorg Med. Chem..

[86] Wortzel I., Seger R. (2011). The ERK Cascade: Distinct Functions within Various Subcellular Organelles. Genes. Cancer.

[87] Sun Y., Liu W.Z., Liu T., Feng X., Yang N., Zhou H.F. (2015). Signaling pathway of MAPK/ERK in cell proliferation, differentiation, migration, senescence and apoptosis. J. Recept. Signal Transduct. Res..

[88] Seger R., Krebs E.G. (1995). The MAPK signaling cascade. FASEB J..

[89] Roskoski R. (2012). ERK1/2 MAP kinases: Structure, function, and regulation. Pharmacol. Res..

[90] Sanyanusin P., Norrish J.H., Ward T.A., Nebel A., McNoe L.A., Eccles M.R. (1996). Genomic structure of the human PAX2 gene. Genomics.

[91] Baldewijns M.M., van Vlodrop I.J., Vermeulen P.B., Soetekouw P.M., van Engeland M., de Bruine A.P. (2010). VHL and HIF signalling in renal cell carcinogenesis. J. Pathol..

[92] Simon M.A. (2000). Receptor tyrosine kinases: Specific outcomes from general signals. Cell.

[93] Lai S., Pelech S. (2016). Regulatory roles of conserved phosphorylation sites in the activation T-loop of the MAP kinase ERK1. Mol. Biol. Cell.

[94] Flannery S., Bowie A.G. (2010). The interleukin-1 receptor-associated kinases: Critical regulators of innate immune signalling. Biochem. Pharmacol..

[95] Guegan J.P., Fremin C., Baffet G. (2012). The MAPK MEK1/2-ERK1/2 Pathway and Its Implication in Hepatocyte Cell Cycle Control. Int. J. Hepatol..

[96] Vollmer S., Strickson S., Zhang T., Gray N., Lee K.L., Rao V.R., Cohen P. (2017). The mechanism of activation of IRAK1 and IRAK4 by interleukin-1 and Toll-like receptor agonists. Biochem. J..

[97] Chen L., Zheng L., Chen P., Liang G. (2020). Myeloid Differentiation Primary Response Protein 88 (MyD88): The Central Hub of TLR/IL-1R Signaling. J. Med. Chem..

[98] Hsieh C.H., Jian C.Z., Lin L.I., Low G.S., Ou P.Y., Hsu C., Ou D.L. (2022). Potential Role of CXCL13/CXCR5 Signaling in Immune Checkpoint Inhibitor Treatment in Cancer. Cancers.

[99] Wang B., Wang M., Ao D., Wei X. (2022). CXCL13-CXCR5 axis: Regulation in inflammatory diseases and cancer. Biochim. Biophys. Acta Rev. Cancer.

[100] De la Mota-Peynado A., Chernoff J., Beeser A. (2011). Identification of the atypical MAPK Erk3 as a novel substrate for p21-activated kinase (Pak) activity. J. Biol. Chem..

[101] Chen C., Nelson L.J., Avila M.A., Cubero F.J. (2019). Mitogen-Activated Protein Kinases (MAPKs) and Cholangiocarcinoma: The Missing Link. Cells.

[102] Hegazy M., Elkady M.A., Yehia A.M., Elsakka E.G.E., Abulsoud A.I., Abdelmaksoud N.M., Elshafei A., Abdelghany T.M., Elkhawaga S.Y., Ismail A. (2023). The role of miRNAs in laryngeal cancer pathogenesis and therapeutic resistance—A focus on signaling pathways interplay. Pathol. Res. Pract..

[103] McKay M.M., Morrison D.K. (2007). Integrating signals from RTKs to ERK/MAPK. Oncogene.

[104] Cargnello M., Roux P.P. (2011). Activation and function of the MAPKs and their substrates, the MAPK-activated protein kinases. Microbiol. Mol. Biol. Rev..

[105] Lau A.T.Y., Xu Y.M. (2018). Regulation of human mitogen-activated protein kinase 15 (extracellular signal-regulated kinase 7/8) and its functions: A recent update. J. Cell Physiol..

[106] Dietz K.J., Jacquot J.P., Harris G. (2010). Hubs and bottlenecks in plant molecular signalling networks. New Phytol..

[107] Bogucka K., Pompaiah M., Marini F., Binder H., Harms G., Kaulich M., Klein M., Michel C., Radsak M.P., Rosigkeit S. (2020). ERK3/MAPK6 controls IL-8 production and chemotaxis. Elife.

[108] Xiang W., Chao Z.Y., Feng D.Y. (2015). Role of Toll-like receptor/MYD88 signaling in neurodegenerative diseases. Rev. Neurosci..

[109] Morris R., Kershaw N.J., Babon J.J. (2018). The molecular details of cytokine signaling via the JAK/STAT pathway. Protein Sci..

[110] Ribas C., Penela P., Murga C., Salcedo A., Garcia-Hoz C., Jurado-Pueyo M., Aymerich I., Mayor F. (2007). The G protein-coupled receptor kinase (GRK) interactome: Role of GRKs in GPCR regulation and signaling. Biochim. Biophys. Acta.

[111] Gurevich V.V., Gurevich E.V. (2019). GPCR Signaling Regulation: The Role of GRKs and Arrestins. Front. Pharmacol..

[112] Tripathi D.K., Poluri K.M. (2020). Molecular insights into kinase mediated signaling pathways of chemokines and their cognate G protein coupled receptors. Front. Biosci..

[113] Tao J., Jiang C., Guo P., Chen H., Zhu Z., Su T., Zhou W., Tai Y., Han C., Ma Y. (2023). A novel GRK2 inhibitor alleviates experimental arthritis through restraining Th17 cell differentiation. Biomed. Pharmacother..

[114] Tian T., Wei M., Guan Y., Rao L., Luo T., Han C., Wei W., Ma Y. (2025). Paeoniflorin-6′-O-benzene sulfonate inhibits keratinocyte proliferation by restoring GRK2-JAK1 colocalization in mouse model of psoriasis. Cell Signal.

[115] Palikhe S., Ohashi W., Sakamoto T., Hattori K., Kawakami M., Andoh T., Yamazaki H., Hattori Y. (2019). Regulatory Role of GRK2 in the TLR Signaling-Mediated iNOS Induction Pathway in Microglial Cells. Front. Pharmacol..

[116] Zarubin T., Han J. (2005). Activation and signaling of the p38 MAP kinase pathway. Cell Res..

[117] Roux P.P., Blenis J. (2004). ERK and p38 MAPK-activated protein kinases: A family of protein kinases with diverse biological functions. Microbiol. Mol. Biol. Rev..

[118] Yoshimura A., Naka T., Kubo M. (2007). SOCS proteins, cytokine signalling and immune regulation. Nat. Rev. Immunol..

[119] Liang Y.B., Tang H., Chen Z.B., Zeng L.J., Wu J.G., Yang W., Li Z.Y., Ma Z.F. (2017). Downregulated SOCS1 expression activates the JAK1/STAT1 pathway and promotes polarization of macrophages into M1 type. Mol. Med. Rep..

[120] Rane S.G., Reddy E.P. (2000). Janus kinases: Components of multiple signaling pathways. Oncogene.

[121] Bousoik E., Montazeri Aliabadi H. (2018). “Do We Know Jack” About JAK? A Closer Look at JAK/STAT Signaling Pathway. Front. Oncol..

[122] Rawlings J.S., Rosler K.M., Harrison D.A. (2004). The JAK/STAT signaling pathway. J. Cell Sci..

[123] Gorina R., Font-Nieves M., Marquez-Kisinousky L., Santalucia T., Planas A.M. (2011). Astrocyte TLR4 activation induces a proinflammatory environment through the interplay between MyD88-dependent NFkappaB signaling, MAPK, and Jak1/Stat1 pathways. Glia.

[124] Liau N.P.D., Laktyushin A., Lucet I.S., Murphy J.M., Yao S., Whitlock E., Callaghan K., Nicola N.A., Kershaw N.J., Babon J.J. (2018). The molecular basis of JAK/STAT inhibition by SOCS1. Nat. Commun..

[125] Ke R., Xu Q., Li C., Luo L., Huang D. (2018). Mechanisms of AMPK in the maintenance of ATP balance during energy metabolism. Cell Biol. Int..

[126] Findlay G.M., Harrington L.S., Lamb R.F. (2005). TSC1-2 tumour suppressor and regulation of mTOR signalling: Linking cell growth and proliferation?. Curr. Opin. Genet. Dev..

[127] Chen W., Pan Y., Wang S., Liu Y., Chen G., Zhou L., Ni W., Wang A., Lu Y. (2017). Cryptotanshinone activates AMPK-TSC2 axis leading to inhibition of mTORC1 signaling in cancer cells. BMC Cancer.

[128] Lin H.P., Lin C.Y., Huo C., Jan Y.J., Tseng J.C., Jiang S.S., Kuo Y.Y., Chen S.C., Wang C.T., Chan T.M. (2015). AKT3 promotes prostate cancer proliferation cells through regulation of Akt, B-Raf, and TSC1/TSC2. Oncotarget.

[129] Sharma A., Mehan S. (2021). Targeting PI3K-AKT/mTOR signaling in the prevention of autism. Neurochem. Int..

[130] Lang F., Cohen P. (2001). Regulation and physiological roles of serum- and glucocorticoid-induced protein kinase isoforms. Sci. STKE.

[131] Palmada M., Speil A., Jeyaraj S., Bohmer C., Lang F. (2005). The serine/threonine kinases SGK1, 3 and PKB stimulate the amino acid transporter ASCT2. Biochem. Biophys. Res. Commun..

[132] Efeyan A., Sabatini D.M. (2010). mTOR and cancer: Many loops in one pathway. Curr. Opin. Cell Biol..

[133] Bago R., Sommer E., Castel P., Crafter C., Bailey F.P., Shpiro N., Baselga J., Cross D., Eyers P.A., Alessi D.R. (2016). The hVps34-SGK3 pathway alleviates sustained PI3K/Akt inhibition by stimulating mTORC1 and tumour growth. EMBO J..

[134] Nobukini T., Thomas G. (2004). The mTOR/S6K signalling pathway: The role of the TSC1/2 tumour suppressor complex and the proto-oncogene Rheb. Novartis Found. Symp..

[135] Yang Q., Inoki K., Kim E., Guan K.L. (2006). TSC1/TSC2 and Rheb have different effects on TORC1 and TORC2 activity. Proc. Natl. Acad. Sci. USA.

[136] Ugi S., Imamura T., Maegawa H., Egawa K., Yoshizaki T., Shi K., Obata T., Ebina Y., Kashiwagi A., Olefsky J.M. (2004). Protein phosphatase 2A negatively regulates insulin’s metabolic signaling pathway by inhibiting Akt (protein kinase B) activity in 3T3-L1 adipocytes. Mol. Cell Biol..

[137] Andrabi S., Gjoerup O.V., Kean J.A., Roberts T.M., Schaffhausen B. (2007). Protein phosphatase 2A regulates life and death decisions via Akt in a context-dependent manner. Proc. Natl. Acad. Sci. USA.

[138] Bao F., Hao P., An S., Yang Y., Liu Y., Hao Q., Ejaz M., Guo X.X., Xu T.R. (2021). Akt scaffold proteins: The key to controlling specificity of Akt signaling. Am. J. Physiol. Cell Physiol..

[139] Schmidt M.F. (2017). miRNA Targeting Drugs: The Next Blockbusters?. Methods Mol. Biol..

[140] Bonneau E., Neveu B., Kostantin E., Tsongalis G.J., De Guire V. (2019). How close are miRNAs from clinical practice? A perspective on the diagnostic and therapeutic market. Ejifcc.

[141] Sun X., Setrerrahmane S., Li C., Hu J., Xu H. (2024). Nucleic acid drugs: Recent progress and future perspectives. Signal Transduct. Target. Ther..

[142] Chakraborty C., Sharma A.R., Sharma G., Lee S.S. (2021). Therapeutic advances of miRNAs: A preclinical and clinical update. J. Adv. Res..

[143] Holjencin C., Jakymiw A. (2022). MicroRNAs and Their Big Therapeutic Impacts: Delivery Strategies for Cancer Intervention. Cells.

[144] Kim T., Croce C.M. (2023). MicroRNA: Trends in clinical trials of cancer diagnosis and therapy strategies. Exp. Mol. Med..

